# TNMD BRICHOS domain attenuates tau pathology and memory deficits in a mouse model of tauopathy

**DOI:** 10.1038/s41419-026-08749-3

**Published:** 2026-04-24

**Authors:** Dandan Su, Huina Li, Lina Pan, Honggui Huang, Tingting Xiao, Guiqin Chen, Wei Tan, Lihong Bu, Zhentao Zhang

**Affiliations:** 1 Department of Neurology, Renmin Hospital of Wuhan University, Wuhan, China; 2 The Central Laboratory, Renmin Hospital of Wuhan University, Wuhan, China; 3 Geriatric Hospital Affiliated to Wuhan University of Science and Technology, Wuhan, China; 4 PET-CT/MRI Center, Faculty of Radiology and Nuclear Medicine, Renmin Hospital of Wuhan University, Wuhan, China; 5 TaiKang Center for Life and Medical Sciences, Wuhan University, Wuhan, China

**Keywords:** Learning and memory, Synaptic transmission

## Abstract

The aberrant aggregation of tau leads to loss of its physiological functions and gain of toxic functions, and plays a crucial role in the pathogenesis of tauopathies including Alzheimer’s disease (AD). Targeting tau aggregation is considered a promising strategy for treating tauopathies. The BRICHOS family consists of a variety of proteins containing the BRICHOS domain. Certain endogenous BRICHOS domains may inhibit the pathological aggregation of disease-associated proteins. However, the effects of the BRICHOS domains on tau aggregation remain unknown. Here we revealed that BRICHOS domains from integral membrane protein 2B (ITM2B), tenomodulin (TNMD), and out at first (OAF) bind to tau and inhibit its aggregation in vitro. Intravenous administration of TNMD BRICHOS alleviates tau aggregation, synaptic dysfunction, and memory deficits in Tau P301S transgenic mice. Thus, TNMD BRICHOS may serve as a potential therapeutic approach for the development of treatments for tauopathies.

## Introduction

Tau was initially characterized as a microtubule-binding protein that is expressed predominantly in neurons and is highly soluble. However, tau is extensively localized within insoluble inclusions in tauopathies, including Alzheimer’s disease (AD) and frontotemporal dementia (FTD)[1]. Extensive research has demonstrated a robust association between tau pathology and cognitive dysfunction[2, 3]. Diminishing tau aggregation may represent a promising strategy to treat tauopathies. Both small-molecule compounds and immunotherapies have been developed to specifically target tau aggregation[4, 5]. Nevertheless, the majority have shown limited efficacy in trials, with a few candidates still under investigation[5–8]. Moreover, several critical challenges remain to be addressed, including ensuring safety, enhancing blood-brain barrier penetration, and overcoming other formulation-related hurdles[5, 9]. Recently, it has been reported that some D-peptides prevent tau aggregation and fragment tau fibrils to neutralize their seeding ability[10, 11]. Thus, peptides may represent a therapeutic approach for treating tauopathies.

BRICHOS-containing proteins constitute a superfamily spanning 11 metazoan phyla, encompassing over 3000 proteins. These proteins share a conserved architecture comprising an N-terminus with a hydrophobic transmembrane (TM) region and a cytoplasmic domain or signal peptide, a linker region, a BRICHOS domain, and a C-terminal domain with a high β-strand propensity[12]. To date, at least ten BRICHOS-containing proteins have been identified in humans, including ITM2A, ITM2B, ITM2C, gastrokine-1 (GKN1), GKN2, TNMD, chondromodulin (CNMD), precursor protein pulmonary-associated surfactant protein C (ProSP-C), BRICHOS-containing domain 5 (BRICD5) and out at first protein (OAF)[12–14]. The BRICHOS domains function as molecular chaperones, inhibiting the aggregation of the C-terminal domain to preserve its normal function[12]. In addition, certain BRICHOS domains have been shown to inhibit the aggregation of disease-associated proteins, such as amyloid-β (Aβ) and islet amyloid polypeptide (IAPP)[15–18]. Research on the BRICHOS domain has focused primarily on ITM2B, ITM2C, and proSP-C, with limited knowledge on other members[15, 19, 20]. The influence of the BRICHOS domain on tau pathology remains unknown.

Here, we screened BRICHOS domains that can inhibit tau aggregation. We found that the BRICHOS domains of ITM2B, TNMD, and OAF bind tau and inhibit its aggregation in vitro, with TNMD BRICHOS demonstrating the highest efficacy. Furthermore, intravenous administration of TNMD BRICHOS attenuated tau pathology and cognitive dysfunction in Tau P301S transgenic mice, a model of tauopathies.

## Results

### Identifying BRICHOS domains that bind tau

**Fig. 1:**
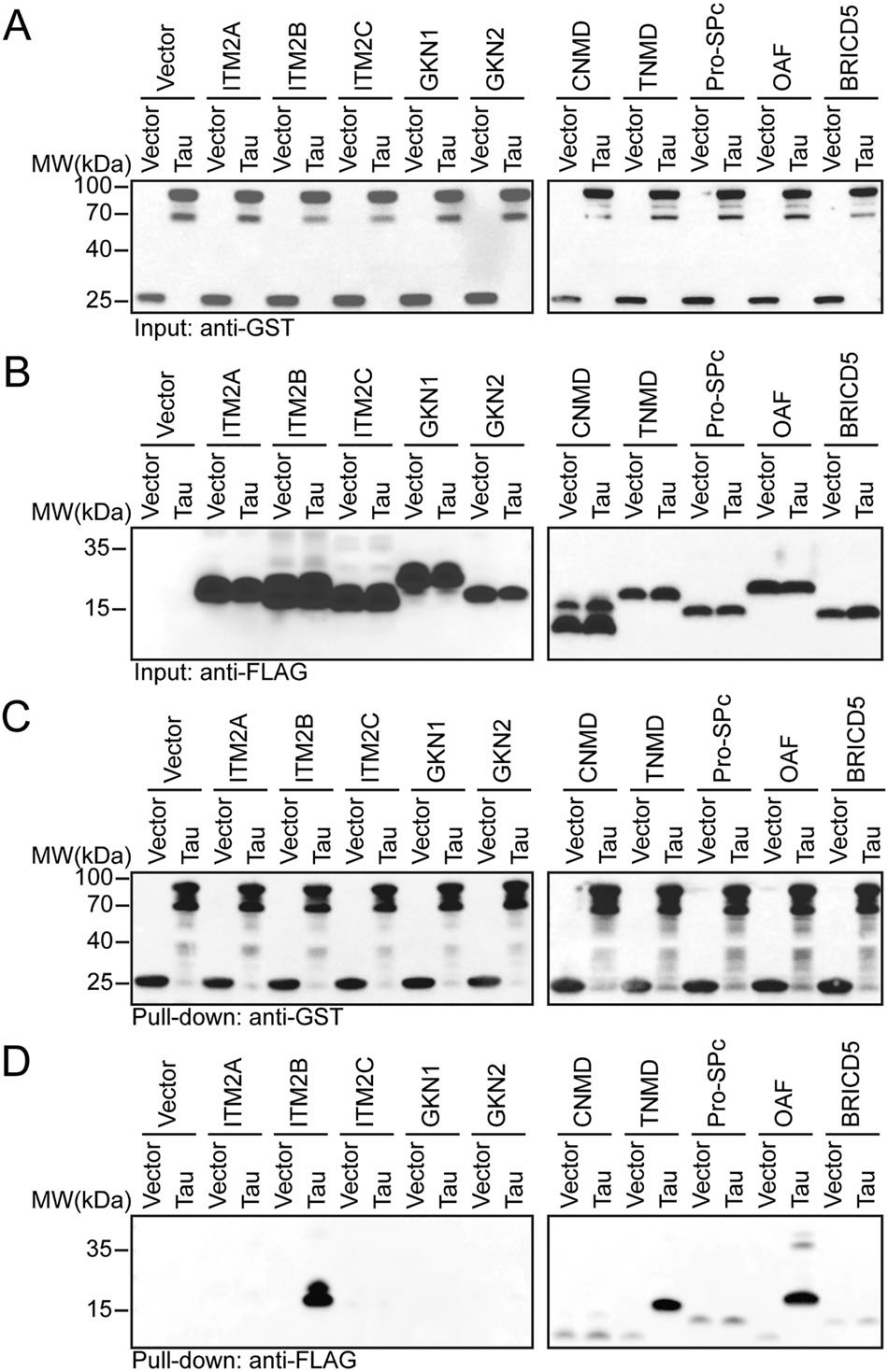
BRICHOS domains from ITM2B, TNMD, and OAF bind tau. HEK293 cells were co-transfected with FLAG-tagged BRICHOS and GST-tagged tau. Shown are representative immunoblots of GST and FLAG in the cell lysates (**A**, **B**) and the GST pull-down samples (**C**, **D**). Shown are the representative image of three independent experiments.

To identify BRICHOS domains that bind tau, the cDNAs of the BRICHOS domains from 10 BRICHOS-containing proteins expressed by human (ITM2A, ITM2B, ITM2C, GKN1, GKN2, TNMD, CNMD, proSP-C, BRICD5, or OAF) were cloned and inserted into the eukaryotic expression plasmid pcDNA3.1. A FLAG-tag sequence was attached to the N-terminus to facilitate protein detection. HEK293 cells were co-transfected with GST-tagged tau and FLAG-tagged BRICHOS plasmids. The expression of FLAG-tagged BRICHOS and GST-tagged tau was tested at 36 h after transfection (Fig. 1A, B). GST pull-down assays revealed that the BRICHOS domain from ITM2B, TNMD, and OAF binds tau, whereas the other BRICHOS domains do not (Fig. 1C, D).

### BRICHOS domains inhibit tau aggregation in vitro

**Fig. 2:**
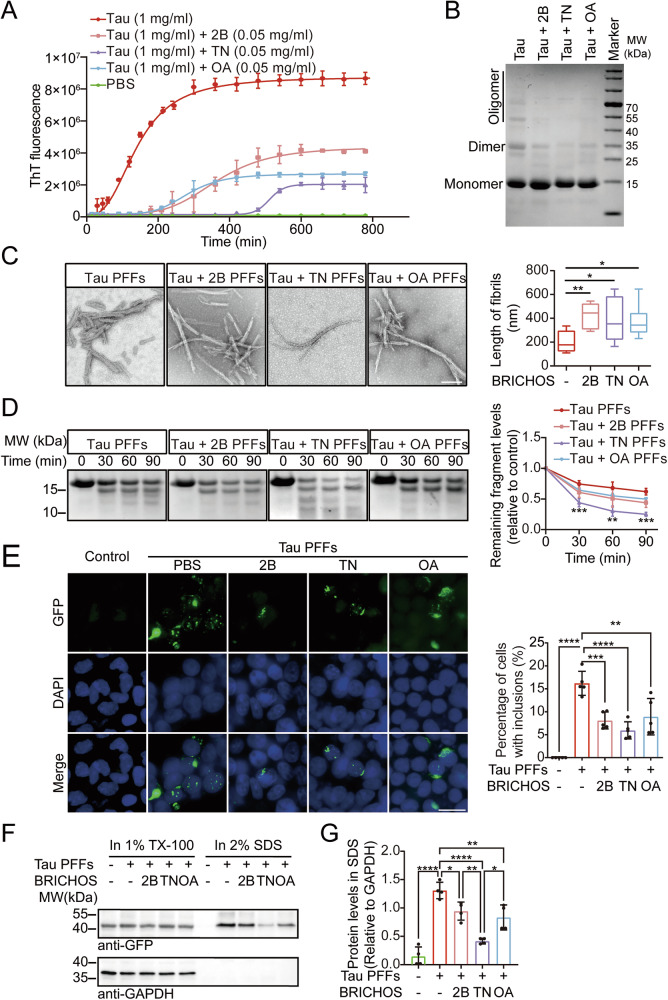
The BRICHOS domains inhibit tau aggregation. **A** Thioflavin T assay showing the kinetics of tau aggregation in the presence or absence of different BRICHOS domains (*n* = 3 independent experiments). **B** Coomassie blue staining showing the aggregation of tau in the presence or absence of BRICHOS domains. **C** Electron microscopy images showing the morphology of the tau fibrils formed in the presence or absence of BRICHOS domains. Scale bar = 0.2 μm. Right panel: quantification of fiber length (*n* = 8 fibrils per group). **D** Protease K (PK) digestion patterns of tau PFFs formed in the presence or absence of BRICHOS domains. PFFs were incubated with 2 µg/ml PK for 30, 60, or 90 min. Right panel: quantitation of the remaining protein (*n* = 3 independent experiments). **E** Tau RD-GFP cells were incubated with recombinant BRICHOS domains (0.25 μg/ml) or PBS and then transfected with PBS or tau PFFs (5 μg/ml). Scale bar = 20 μm. Right panel: quantitation of the percentage of cells with tau inclusions (*n* = 5 independent experiments). **F** Sequential extraction experiments. Tau RD-GFP cells transfected with Tau PFFs were extracted with 1% Triton X-100 (TX-100) lysis buffer followed by 2% SDS lysis buffer. The cell lysates from each fraction were immunoblotted with anti-GFP antibodies. **G** Quantitation of the levels of GFP in the SDS-soluble fraction (relative to the level of GAPDH in the 1% TX-100 fraction) (*n* = 5 independent experiments). Data are presented as mean ± SD (**A**, **D**, **E**, **G**) or box plots (**C**) (centerlines, medians; box limits, upper and lower quartiles; whiskers, min to max). *P-*values were determined by Kruskal–Wallis test followed by Dunn’s multiple comparisons (**C**), two-way ANOVA with Dunnett’s multiple comparisons test (**D**) or one-way ANOVA with Tukey’s multiple comparisons test (**E**, **G**). *P < 0.05, **P < 0.01, ***P < 0.001, ****P < 0.0001. 2B ITM2B-BRICHOS, TN TNMD-BRICHOS, OA OAF-BRICHOS.

To investigate the effects of the BRICHOS domains from ITM2B, TNMD, and OAF on tau aggregation, we purified these BRICHOS domains and tau K18 fragment. K18 was incubated in the presence or absence of the BRICHOS domains. Thioflavin T (ThT) assays revealed that all three BRICHOS domains suppressed tau aggregation (Fig. 2A). The aggregation of tau was further confirmed by Coomassie blue staining, in which fewer high-molecular-weight K18 species were found in the K18 solution in the presence of BRICHOS (Fig. 2B). Transmission electron microscopy (TEM) analysis revealed that different morphologies of tau PFFs formed in the presence of BRICHOS domains. The tau fibrils formed in the presence of BRICHOS domains presented longer fibers (Fig. 2C). Furthermore, the tau fibrils formed in the presence of BRICHOS exhibited reduced resistance to proteinase K (PK) digestion compared with the tau fibrils formed in the absence of BRICHOS domains. TNMD BRICHOS had the most dramatic effect on PK digestion (Fig. 2D). We also performed molecular docking simulations between BRICHOS and tau RD with AlphaFold. It supports that there may be an interaction between BRICHOS and tau RD, and TNMD BRICHOS possesses more binding sites for tau RD. (Supplementary Fig. 1A–C). These results indicate that the BRICHOS domains inhibit the assembly of tau into filaments in vitro.

To test whether the BRICHOS domain inhibits tau aggregation in cells, we used HEK293 cell lines stably expressing the tau repeat domain (Tau RD-GFP cells). These cells develop tau inclusions when exposed to exogenous preformed fibrils (PFFs), serving as a cellular model of tau aggregation[21]. The cells were exposed to recombinant BRICHOS and then transfected with tau PFFs. Forty-eight hours after transfection, BRICHOS signals were detected in the cells (Supplementary Fig. 2A). Interestingly, fewer aggregates were observed in cells pre-treated with the BRICHOS domains (Fig. 2E). Next, we performed sequential extraction of cell lysates to test the levels of insoluble tau. Consistently, Triton X-100-insoluble tau species were observed in cells treated with tau PFFs, and this effect was attenuated by pre-treatment with BRICHOS domains (Fig. 2F, G). These results indicate that the BRICHOS domain attenuates tau aggregation in vitro.

### BRICHOS domains attenuate tau aggregation and toxicity in primary neurons

**Fig. 3:**
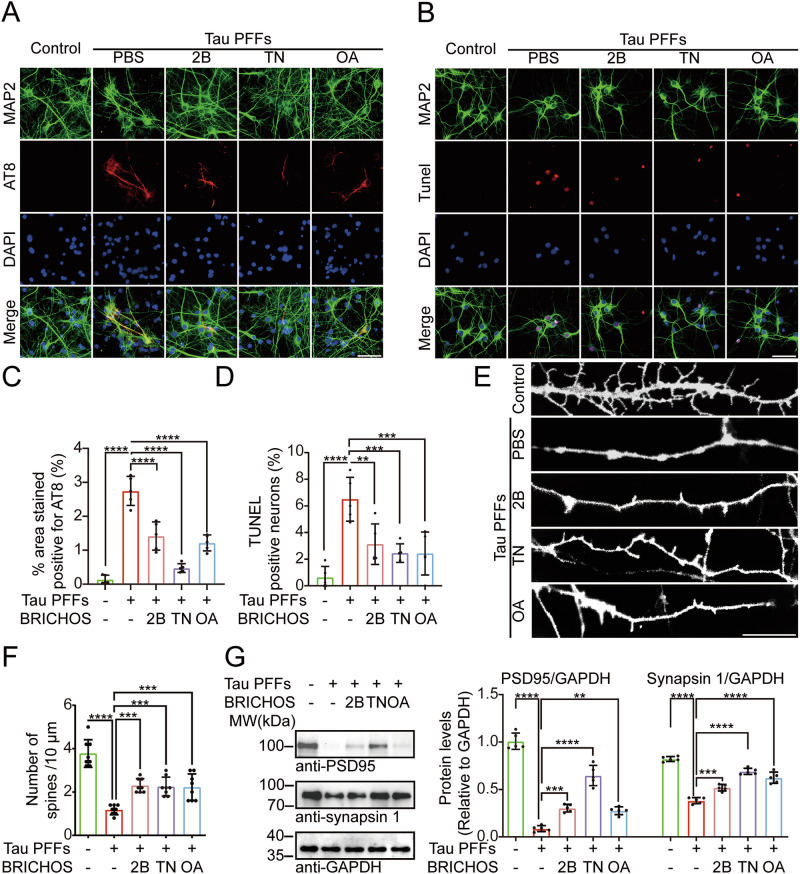
BRICHOS domains suppress tau phosphorylation and neurotoxicity in primary neurons. **A**, **B** Primary neurons were treated with BRICHOS domains (0.25 μg/ml) or PBS and then treated with PBS or tau PFFs (5 μg/ml). The primary neurons were subjected to double immunofluorescence staining for MAP2 and AT8 (**A**) or TUNEL (**B**). Scale bar = 50 μm. Quantification of AT8-positive areas (**C**) or TUNEL-positive neurons (**D**) (*n* = 6 independent experiments). **E** Representative images of DiI-stained dendritic spines from different groups. Scale bar = 10 μm. **F** Quantitation of the density of spines (*n* = 8 independent experiments). **G** Lysates of primary neurons in different groups were immunoblotted with synapsin 1 and PSD95 antibodies. Right panel: quantification of the levels of synapsin 1 and PSD95 (*n* = 5 independent experiments). Data are presented as means ± SD. *P*-values were determined by one-way ANOVA with Tukey’s multiple comparisons test. *P < 0.05, **P < 0.01, ***P < 0.001, ****P < 0.0001. 2B ITM2B-BRICHOS, TN TNMD-BRICHOS, OA OAF-BRICHOS.

We further determined whether the BRICHOS domains relieve neurotoxicity in primary neurons. The neurons were treated with recombinant BRICHOS and then with tau PFFs (5 μg/ml final concentration) for 5 days. BRICHOS were detected in neurons (Supplementary Fig. 2B). The phosphorylation of endogenous tau was analyzed by immunostaining with the AT8 antibody. The results revealed that treatment with tau PFFs promoted tau phosphorylation, whereas pre-treatment with the BRICHOS domain alleviated tau phosphorylation (Fig. 3A, C). TUNEL staining revealed that tau PFFs induced neuronal apoptosis, which was alleviated by pre-treatment with the BRICHOS domain (Fig. 3B, D). We further assessed the density of dendritic spines along individual dendrites using DiI staining. Tau PFFs induced the loss of dendritic spines, which was partially alleviated by pre-treatment with the BRICHOS domains (Fig. 3E, F). Furthermore, BRICHOS attenuated the loss of the synaptic marker proteins PSD95 and synapsin 1 induced by tau PFFs (Fig. 3G). Collectively, these results suggest that BRICHOS effectively alleviates tau aggregation and neurotoxicity in primary neurons.

### TNMD BRICHOS crosses the blood-brain barrier

**Fig. 4:**
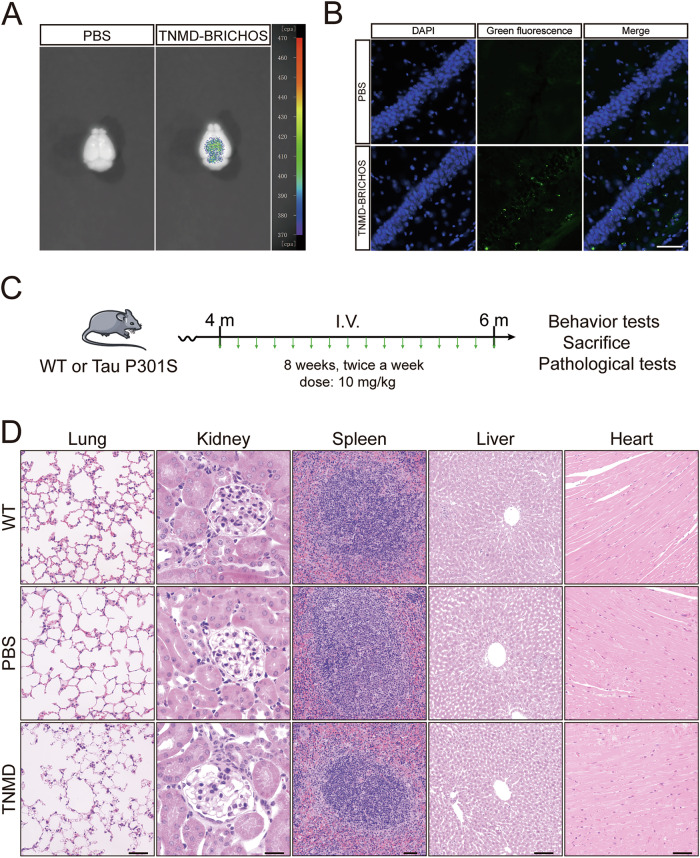
TNMD BRICHOS passes the blood-brain barrier. **A** Wild-type mice were administered fluorescent-labeled TNMD BRICHOS (10 mg/kg) via the tail vein. One hour later, the mice were perfused to wash out residual TNMD BRICHOS from the blood vessels. Fluorescence signals were tested in a small animal imaging system. **B** Florescence images showing the presence of TNMD BRICHOS (green) in the CA1 area. The nuclei were stained with DAPI (blue). Scale bar = 100 μm. **C** Diagram showing the process of the animal experiments. **D** Representative H&E staining of the heart, liver, spleen, lung, and kidney of mice from each group. Scale bar of lung, kidney, spleen, and heart = 50 μm. Scale bar of liver = 100 μm. Shown are the representative image of 3 mice/group.

Since TNMD BRICHOS demonstrated favorable efficacy in the in vitro experiments, we further tested its efficacy in animal models. Three-month-old wild-type mice were intravenously injected with fluorescently labeled TNMD BRICHOS or vehicle. One hour later, the mice were euthanized and perfused with PBS to wash out residual TNMD BRICHOS from the blood vessels. TNMD BRICHOS signals were detected using a small animal in vivo imaging system, indicating that it crosses the blood-brain barrier (Fig. 4A). Consistently, fluorescence images of brain sections showed that the peptide was detectable in various brain areas, including the hippocampus (CA1, DG, CA2 and CA3), entorhinal cortex and prefrontal cortex (Fig. 4B and Supplementary Fig. 3A). Furthermore, the peptide signals colocalized with the neuronal marker MAP2, implying that these peptides are internalized by neurons (Supplementary Fig. 3B). Four-month-old tau P301S mice or WT mice were injected with recombinant TNMD BRICHOS (10 mg/kg) or PBS via the tail vein twice weekly for 2 months (Fig. 4C). Hematoxylin-eosin (HE) staining of major organs (hearts, livers, spleens, lungs, and kidneys) revealed no obvious histopathological changes (Fig. 4D). These data suggest that chronic treatment with TNMD-BRICHOS at a dosage of 10 mg/kg does not result in systemic toxicity in mice.

### TNMD BRICHOS attenuates cognitive impairments in Tau P301S mice

**Fig. 5:**
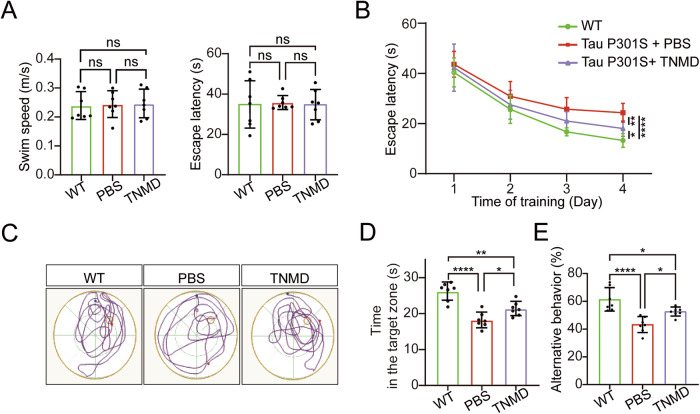
TNMD BRICHOS alleviates memory deficits in Tau P301S mice. **A** The swimming speed of the mice and the time spent on the platform during visible training in the Morris water maze test. **B** Escape latency to reach the platform during hidden platform training. **C** Representative movement trajectories of the mice in the probe trial. Blue marks the starting point of the mouse’s movement, while red indicates the endpoint. **D** Time spent in the target quadrant in the probe trial. **E** Spontaneous alternation behavior scores in the Y-maze test (*n* = 7 mice/group). Data are presented as means ± SD. *P*-values were determined by one-way ANOVA with Tukey’s multiple comparisons test. *P < 0.05, **P < 0.01, ****P < 0.0001, ns not significant.

To further investigate whether TNMD BRICHOS could rescue memory deficits in Tau P301S mice, we conducted Morris water maze (MWM) and Y-maze tests. On the first day of the visible platform test in the MWM test, the mice in the different groups had similar swimming speeds and spent similar amounts of time reaching the platform, indicating that the visual and motor abilities of the mice were not affected by the injection of TNMD BRICHOS (Fig. 5A). During the training phase, vehicle-treated Tau P301S mice took a longer time to reach the platform than did WT mice, which was alleviated in Tau P301S mice injected with TNMD BRICHOS (Fig. 5B). In the probe trial, the Tau P301S mice spent less time in the quadrant where the original platform was located. The time was longer in TNMD BRICHOS-injected Tau P301S mice than in vehicle-injected Tau P301S mice (Fig. 5C, D). Consistently, in the Y-maze test, the percentage of spontaneous alternations in vehicle-injected Tau P301S mice was lower than that in WT mice, indicating impaired special memory. This impairment was attenuated by the injection of TNMD BRICHOS (Fig. 5E). These data suggest that intravenous injection of TNMD BRICHOS rescues memory deficits in Tau P301S transgenic mice.

### TNMD BRICHOS ameliorates tau phosphorylation and synaptic degeneration in Tau P301S mice

**Fig. 6:**
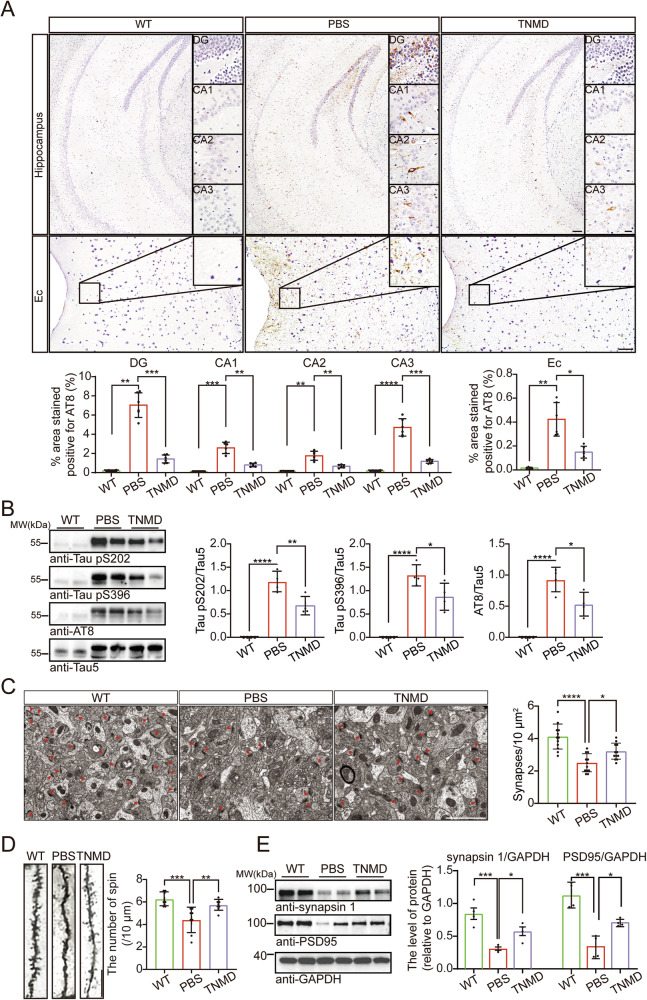
TNMD BRICHOS alleviates tau pathology and synaptic degeneration in Tau P301S mice. **A** Representative images of AT8-positive tau pathology in the hippocampus dentate gyrus (DG), CA1, CA2, CA3 and entorhinal cortex (Ec). Scale bar of hippocampus = 100 μm. Scale bar of DG, CA1, CA2, CA3 = 25 μm. Scale bar of entorhinal cortex = 50 μm. Lower panel: Quantification of AT8-positive areas (*n* = 6 images/group). **B** Representative immunoblots of p-tau (S202, S396, AT8) and total tau (tau5) in the hippocampus. Right panel: quantification of S202, S396, and AT8 (*n* = 4 mice/group). **C** Electron microscopy images of synapses in the hippocampus. Red arrows indicate synaptic structures. Scale bar = 20 μm. Right panel: quantification of synaptic density (*n* = 12 images/group). **D** Golgi staining images of dendritic spines in the hippocampal region. Scale bar = 10 μm. Right panel: quantification of dendritic spine density (*n* = 8 neurons/group). **E** Western blotting of synaptic markers (synapsin 1 and PSD95) in the hippocampus. Right panel: quantification of synapsin 1 and PSD95 protein levels (*n* = 4 mice/group). Data are presented as means ± SD. *P*-values were determined by Brown-Forsythe ANOVA with Dunnett’s T3 multiple comparisons test (**A**) or one-way ANOVA with Tukey’s multiple comparisons test (**B**–**E**). *P < 0.05, **P < 0.01, ***P < 0.001, ****P < 0.0001.

We further investigated the level of tau phosphorylation in the mouse brain. Immunohistochemical staining revealed that tau phosphorylation in the hippocampus and entorhinal cortex was alleviated by intravenous injection of TNMD BRICHOS (Fig. 6A). Western blot analysis confirmed that tau phosphorylation was attenuated by TNMD BRICHOS (Fig. 6B). These results indicate that intravenous injections of TNMD BRICHOS inhibit tau phosphorylation in vivo. Synaptic dysfunction represents one of the early pathological features of tauopathies[22, 23]. Transmission electron microscopy was used to evaluate the density of synapses in the hippocampal region. Compared with that of WT mice, the synaptic density of the hippocampus of Tau P301S transgenic mice was decreased, which was partially attenuated by TNMD BRICHOS (Fig. 6C). Golgi staining of dendritic spines revealed that TNMD BRICHOS attenuated the loss of dendritic spines in the hippocampal area (Fig. 6D). Consistently, the levels of synaptic markers, including PSD95 and synapsin 1, in the hippocampus were decreased in vehicle-injected Tau P301S mice and attenuated by TNMD BRICHOS (Fig. 6E). Collectively, these data indicate that intravenous injection of TNMD BRICHOS ameliorates tau phosphorylation and the synaptic degeneration of Tau P301S transgenic mice.

## Discussion

In this study, we screened BRICHOS domains that can inhibit tau aggregation. We found that the TNMD BRICHOS domain has favorable effects both in vitro and in vivo. Intravenous injection of TNMD BRICHOS alleviates tau phosphorylation, synaptic loss, and memory impairments in Tau P301S mice. These results suggest that the TNMD BRICHOS domain may hold promise as a potential therapeutic approach for tauopathies.

Tau is expressed mainly in axons, where it plays a key role in maintaining the stability of microtubules[24]. Under pathological conditions, tau undergoes excessive phosphorylation and aggregation, leading to loss of its physiological function and the production of neurofibrillary tangles (NFTs)[24]. Furthermore, tau fibrils can seed the aggregation of tau monomers, resulting in the propagation of tau fibrils[25]. Higher seeding activity of tau is associated with worse cognitive function in patients[26, 27]. Tau aggregates exhibit neurotoxicity, inducing neuronal apoptosis and synaptic dysfunction, which may underlie the positive correlation between tau pathology severity and cognitive impairment[28, 29]. Inhibiting tau aggregation and spread may slow cognitive decline[30, 31]. To eliminate pathological tau and block the intercellular transmission of tau fibrils, several compounds have been developed[32–35]. However, they did not demonstrate a beneficial effect in clinical trials[36]. In this study, we found that TNMD BRICHOS inhibits tau aggregation, ameliorates synapse dysfunction and rescues memory deficits in mice.

TNMD is classified as a type II transmembrane glycoprotein and is highly expressed in tendons[37]. Some studies have shown that TNMD is related to obesity and inflammation[38, 39]. However, the exact physiological and pathological roles of TNMD in the brain remain unknown. Interestingly, single-nucleotide polymorphisms of TNMD are correlated with the APOE ε4 allele, a risk gene for AD[40]. Our findings demonstrate that the TNMD BRICHOS domain exhibits specific binding affinity for tau protein, as evidenced by both pull-down assays and molecular docking simulations. Furthermore, we established through complementary in vitro and in vivo experiments that the TNMD BRICHOS domain effectively suppresses tau aggregation. Although the BRICHOS effects of ITM2B and OAF were not tested in vivo, they demonstrated promising anti-tau protein aggregation effects in vitro. Recent studies have also reported the anti-tau aggregation capability of ITM2B BRICHOS[41]. ITM2B is highly expressed in the brain, and two mutations in the ITM2B gene have been identified as the cause of familial British and Danish dementia[42]. OAF is highly expressed in astrocytes[43]. Its function is related to neuronal development and hatching in *Drosophila*[14]. These results suggest that further research on ITM2B and OAF is warranted.

We identified three BRICHOS domains that can bind to the tau protein. However, the primary sequences of these BRICHOS domains are not similar (Supplementary Fig. 4). Interestingly, the BRICHOS domains of different proteins share similar secondary structures, with a central β-sheet flanked by one α-helix on each side[12]. The solvent exposure of face A, defined as the β-sheet surface facing α-helix 1 in the BRICHOS domain of ITM2B and pro SP-C, is different[44]. Previous studies have demonstrated that Face A plays a pivotal role in the ability of BRICHOS to inhibit the aggregation of disordered proteins[44]. Further comparison of secondary or higher-order structures may facilitate the development of peptides that target tauopathies. In addition, determining the binding interface between BRICHOS and tau is important. Small molecules or antibodies based on structural design could be developed to mimic BRICHOS functions while exhibiting enhanced advantages in target specificity, druggability, modifiability, and safety.

Overall, we found that TNMD BRICHOS can inhibit tau protein aggregation and alleviate cognitive dysfunction in Tau P301S mice. This study has several limitations. One is the reliance on a single tauopathy model (Tau P301S mice), which may not fully recapitulate human sporadic tauopathies. Moreover, the 8-week treatment duration might underestimate long-term effects, as tau pathology in human progresses over several decades. Future work should incorporate other models of tauopathy and longitudinal designs to address these gaps.

## Materials and methods

### Mice

C57BL/6J and Tau P301S transgenic mice (line PS19) were obtained from the Jackson Laboratory (stock numbers: 000664 and 008169, respectively). The mice were kept under specific pathogen–free (SPF) conditions, with the environmental temperature set at 22 °C. They were provided *ad libitum* access to food and water and were subjected to a 12-h light/12-h dark cycle daily. In subsequent animal experiments (where grouping is involved), all animals will be assigned to different groups using the random number table method. Investigators were blinded to the group allocation during the animal experiments. The experimental procedures were approved by the Institutional Animal Care and Use Committee (IACUC) of Renmin Hospital of Wuhan University, with the IACUC issue number (Renmin Hospital of Wuhan University (WDRM) animal (welfare)) 20250102D.

### Plasmid construction

The cDNA of the BRICHOS domain from 10 human-expressed BRICHOS proteins was cloned and inserted into the eukaryotic expression plasmid pcDNA3.1, with each clone having a FLAG tag (DYKDDDDK) at the N-terminus of the BRICHOS domain[45]. To maintain the integrity of the BRICHOS domains, we referred to the literature and extended the predicted protein fragments[13–15]. The specific sequences are detailed in Table 1. The primer information is listed in Supplementary Table 1.

**Table 1:** Amino acid sequences of 10 BRICHOS proteins.

Name	Amino acid sequences
*ITM2A-BRICHOS*	residues 110-225 of full-length ITM2A (NP_004858.1)
*ITM2B-BRICHOS*	residues 113–231 of full-length ITM2B (NP_068839.1)
*ITM2C-BRICHOS*	residues 112–230 of full-length ITM2C (NP_112188.1)
*GKN1-BRICHOS*	residues 21-150 of full-length GKN1 (NP_062563.4)
*GKN2-BRICHOS*	residues 21-150 of full-length GKN2 (NP_872342.2)
*TNMD-BRICHOS*	residues 70-186 of full-length TNMD (NP_071427.2)
*CNMD-BRICHOS*	residues 90-201 of full-length CNMD (NP_008946.1)
*proSP-C-BRICHOS*	residues 86-197 of full-length proSP-C (NP_003009.2)
*BRICD5-BRICHOS*	residues 85-193 of full-length BRICD5 (NP_872369.2)
*OAF-BRICHOS*	residues 29-172 of full-length OAF (NP_848602.1)

### Purification of recombinant BRICHOS and tau

The BRICHOS domains from ITM2B, TNMD, and OAF were cloned and inserted into the PET28c vector. The plasmids were subsequently transformed into *E. coli* BL21 (DE3) cells. Protein extraction was performed following protocols described previously with some modifications[16]. BL21 cells were cultured in Luria-Bertani medium for 6 h at 37 °C. The temperature was then reduced to 20 °C. Isopropyl-beta-D-thiogalactopyranoside (IPTG) was added to achieve a final concentration of 1 mM, and the mixture was incubated overnight. The cells were collected and centrifuged at 10,000 rpm for 10 min. The resulting precipitate was then placed in a solvent (20 mM Tris-HCl with cocktail, pH = 8) and sonicated at 450 W for 20 min. The sediment was collected via centrifugation at 10,000 rpm for 15 min and dissolved in buffer containing 8 M urea at 4 °C overnight. On the third day, the protein reformation process was followed by overnight dialysis in double-distilled water. The products were lyophilized and stored at −80 °C.

The tau fragment (K18), which comprises all four microtubule-binding repeats (4R) of tau with a molecular weight of 13.7 kDa, is often used to study tau aggregation[46, 47], and its extraction protocol was performed as previously described[48]. To obtain the tau fragment, a truncated form of human tau containing only 4R was fused with an N-terminal His-tag, subcloned, and inserted into the KpnI site of the PRK172 bacterial expression vector. The plasmid was subsequently transformed into *E. coli* BL21 (DE3) cells. The cells were grown overnight on a shaker at 37 °C, sonicated in ice-cold buffer containing 20 mM Tris (pH = 8.0), 500 mM NaCl, and 5 mM imidazole, and centrifuged for 30 min at 12,000 rpm at 4 °C. The supernatant was added to a column pre-equilibrated with Ni-NTA His Bind Resin (MERCK). The column was washed with 10 column volumes of 20 mM Tris (pH = 8.0), 500 mM NaCl, and 15 mM imidazole. The protein was then eluted in the same buffer containing 100 mM imidazole. The purified protein was dialyzed against double-distilled water for 24 h and then lyophilized.

### Preparation of tau PFFs

To prepare tau PFFs, lyophilized tau K18 was dissolved in PBS and centrifuged at 15,000 rpm for 20 min to remove the precipitates. The final concentration of the supernatant was adjusted to 1 mg/ml. To facilitate tau aggregation, 2 mM DTT and 12.5 μM low-molecular-weight heparin were added. The solution was incubated overnight at 37 °C with agitation at 1000 rpm. Thioflavin T (ThT) fluorescence assay was conducted to assess the aggregation process of tau in the presence or absence of BRICHOS (0.05 mg/ml final concentration). Aliquots of 10 μl from the incubation mixture were taken at various time points, diluted with 100 μl of PBS containing 2 mM ThT, and incubated for 10 min at room temperature. The fluorescence was recorded at 450 nm excitation and 510 nm emission wavelengths via a SpectraMax plate reader.

### Cell culture and transfection

HEK293 cells were cultured in HEK293 special media (Procell, CM-0208) supplemented with 100 units/mL penicillin and 100 units/mL streptomycin in a humidified atmosphere containing 5% CO_2_ at 37 °C. The HEK293 cell line stably expressing tau 4R, which spans residues 243-365-RD (Tau-RD-HEK293), was established according to previously described methods[21]. The cells were recently authenticated and tested for mycoplasma contamination. Tau-RD-HEK293 cells are capable of forming tau inclusions upon exposure to tau PFFs, serving as a cellular model for tau aggregation[21]. To transfect tau PFFs into Tau-RD-HEK293 cells, tau PFFs (10 μg) were combined with OptiMEM (Gibco) to a final volume of 100 μl. Ninety-five microliters of OptiMEM and 5 μl of Lipofectamine 2000 (Invitrogen) were mixed in another tube and then added to the PFF tube to a final volume of 200 μl. After incubation for 20 min, the mixture was added to the culture medium to induce tau aggregation. To test the effect of BRICHO domains on tau aggregation, the cells were exposed to recombinant BRICHOS domains (0.25 μg/ml final concentration) 6 h before transfection.

### GST pull-down assay

Plasmids encoding GST-tagged tau and FLAG-tagged BRICHOS were co-transfected into HEK293 cells. The GST-tau (15 μg) plasmids were mixed with FLAG-BRICHOS plasmids (15 μg) in 500 μl of OptiMEM. Lipofectamine-2000 (20 μl) was mixed with another 500 μl of OptiMEM. After the two solutions were mixed and incubated at room temperature for 20 min, the mixture was added to the cell medium of the HEK293 cells. The cells were harvested and lysed in NP40 lysis buffer (Beyotime, P0013F). Thirty-six hours after transfection, the cell lysates were incubated with glutathione agarose overnight at 4 °C. The beads were washed 3 times with phosphate buffer saline (PBS) supplemented with 0.1% Triton X-100 (PBST) and boiled in sodium dodecyl sulfate (SDS) loading buffer for 10 min. The samples were subjected to Western blot analysis using GST and FLAG antibodies.

### Western blotting

Cultured cells or mouse brain tissue were lysed on ice for 30 min in NP40 lysis buffer (Beyotime, P0013F) or RIPA lysis buffer (Beyotime, P0013B) supplemented with protease inhibitor cocktail (Sigma‒Aldrich, P9599) and phosphatase inhibitor (APExBIO, K1015). After centrifugation for 15 min at 12,000 rpm and 4 °C, the protein concentrations of the supernatants were determined using a Pierce BCA protein assay kit (Thermo Fisher). After protein separation by 8–12% SDS‒PAGE, the proteins were transferred to a nitrocellulose membrane. The membranes were blocked with 5% non-fat milk in tris buffered saline (TBS) containing 0.1% Tween 20 (TBST) and then incubated with primary antibodies overnight at 4 °C. The membranes were washed 3 times in TBST and incubated with horseradish peroxidase (HRP)-conjugated anti-mouse or anti-rabbit antibodies for 1 h at room temperature. Immunoreactivity was visualized by enhanced chemiluminescence (ECL) using an ECL Western blotting system (Bio-Rad). Primary antibodies against the following proteins were used: GST (Proteintech, 66,001–2-Ig, 1:1000), FLAG (Proteintech, 66008-4, 1:1000), EGFP-HRP (Proteintech, HRP-66002, 1:1000), GAPDH (Proteintech, 60004-1-Ig, 1:8000), synapsin 1 (Cell Signaling Technology, 2312 s, 1:1000), PSD95 (ABclonal, A13354, 1:1000), AT8 (Thermo Fisher, MN1020, 1:1000), phospho-Tau S202 (Abcam, ab108387, 1:1000), phospho-Tau S396 (Abcam, ab32057, 1:1000) and total Tau (Invitrogen, MA5-12808, 1:1000).

### Transmission electron microscopy

Tau PFFs formed in the presence or absence of BRICHOS were visualized via negative stain transmission electron microscopy (TEM) as described previously[48]. Tau PFFs and tau-BRICHOS mixed PFFs were adsorbed to glow-discharged 400 mesh carbon-coated copper grids for 2 min, quickly washed three times with Tris-HCl buffer (50 mM, pH = 7.4), and floated upon two drops of 0.75% uranyl formate for 30 s. The grids were allowed to dry before imaging on a Phillips CM 120 transmission electron microscope operating at 80 kV. The images were captured and digitized with an ER-80 CCD (8 megapixel) by advanced microscopy techniques.

### Proteinase K (PK) digestion

PK (2 μg/ml final concentration) was added to 100 μg of tau PFFs formed in the presence or absence of BRICHOS and incubated at 37 °C for 0, 30, 60, or 90 min. Then, 0.1 μg of protease inhibitor cocktail was added to stop the reaction. The samples were boiled in SDS loading buffer for 10 min and separated in SDS-PAGE. The bands of the PK digestion were detected by Coomassie blue staining. In brief, the protein gel was sequentially submerged in fixative buffer (50% ethanol and 10% acetic acid in double distilled water) for 15 min, Coomassie blue staining buffer (0.25% R250 Coomassie blue dye solution with 25% ethanol and 8% acetic acid in double distilled water) for 15 min, and elution buffer (25% ethanol and 8% acetic acid in double distilled water) overnight at room temperature.

### Immunohistochemistry (IHC) and immunofluorescence staining

For IHC, after anesthetization, the mice were perfused through the heart with normal saline followed by 4% paraformaldehyde. The mouse brains were harvested and immersed in 4% paraformaldehyde overnight for fixation. The brains were then embedded in paraffin and subsequently sliced into 4 μm-thick paraffin sections. These paraffin sections were dewaxed by sequentially immersion in xylene, 100%, 95%, 85%, 75% alcohol, and ddH_2_O. Next, the samples were incubated in antigen retrieval solution at 94 °C for 20 min. After the sections were allowed to cool naturally, they were incubated in 3% hydrogen peroxide to block endogenous peroxidase activity, blocked in 3% bovine serum albumin (BSA), and then incubated at 4 °C overnight with primary antibody (AT8, 1:500). After being washed, the sections were incubated with secondary antibody (HRP-labeled goat anti-mouse IgG, Origene, PV-6002, 1:1000) at 37 °C for 30 min, followed by DAB (Absin, abs957) staining for visualization. The cell nuclei were counterstained with hematoxylin. After staining, the slides were sequentially immersed in 75%, 85%, 100% alcohol and xylene for dehydration. Finally, the sections were mounted with neutral balsam. For immunofluorescence staining, the cells were fixed and permeabilized with 4% paraformaldehyde containing 1% Triton X-100 for 30 min. After being blocked with 3% BSA for 30 min, the slides were incubated with primary antibodies at 4 °C overnight. Primary antibodies against the following proteins were used: MAP2 (Proteintech, 67015-1-1 g, 1:1000), His (Invitrogen, 710286, 1:100). After the samples were washed with PBS 3 times, Alexa Fluor 488 or 594-conjugated secondary antibodies (ThermoFisher, W11261, 1:500) were applied, and the samples were incubated at room temperature for 2 h. The cells were stained with DAPI solution for 5 min. The images were captured using a fluorescence microscope.

### Sequential extraction

The cells were resuspended in TBS containing 1% Triton X-100 and protease inhibitor, lysed by 10% power sonication 10 times, and then centrifuged at 100,000 × *g* at 4 °C for 30 min. The supernatant was stored as the 1% Triton X-100 soluble fraction. The precipitate was washed with PBS and then dissolved in TBS containing 2% SDS. A Bradford assay (Bio-Rad) was used to normalize the protein concentrations. The samples were tested by Western blotting.

### Primary neuronal culture

Primary cortical neurons were derived at embryonic day 18 as described previously[49]. The neurons were cultured in neurobasal medium (Gibco, 21103049) supplemented with B-27 (Gibco, 17504044), 0.5 mM L-glutamine (Gibco, 35050061), penicillin and streptomycin at 37 °C in 5% CO_2_/95% air. To test the effect of BRICHOS domains and PFFs on the aggregation of endogenous tau, recombinant BRICHOS domains (0.25 μg/ml final concentration) and tau PFFs (5 μg/ml final concentration) were added to the medium and incubated for 5 days. For immunostaining, the neurons were fixed and permeabilized with 4% paraformaldehyde for 30 min.

### DiI staining

DiI crystals (Invitrogen, D282) were carefully added to the slides and incubated for 30 min at room temperature. Then, the DiI powder was removed by washing with PBS. The slides were observed under a confocal microscope (Lecia). To measure neuronal dendritic spine density, 8–10 neurons were randomly selected from each group to calculate the number of secondary dendritic spines of 50 μm spines per neuron.

### Fluorescent labeling of TNMD BRICHOS



Under an argon atmosphere, a 50 mL Schlenk tube equipped with a magnetic stirring bar was charged with **1** (1.40 g, 4.4 mmol, 1.1 equiv), *tert*-butyl 6-bromohexanoate (1.11 g, 4.0 mmol, 1.0 equiv), K_2_CO_3_ (0.66 g, 4.8 mmol, 1.2 equiv) and DMF (20 mL), and the mixture was warmed to 90 °C in an oil bath and stirred for 12 h. Then, the mixture was diluted with saturated NaCl aqueous solution. The aqueous phase was extracted with EA (30 mL × 3). The combined organic phase was dried over Na_2_SO_4_ and filtered. The solvents were removed under reduced pressure, and the residue was purified by silica gel chromatography (DCM:MeOH = 20:1) to yield intermediate **2**. Intermediate 2 and 20 mL of DCM were added to a 50 mL round-bottom flask, and then TFA (1.80 g, 15.0 mmol) was added dropwise. The mixture was stirred at room temperature for 12 h. After the reaction was finished, the solvents were removed under reduced pressure, and the residue was purified by silica gel chromatography (DCM:MeOH = 10:1) to give the desired product **3** (0.67 g, 36% two steps, white solid). ^1^H NMR (400 MHz, CD_3_OD) δ 8.33 (d, *J* = 7.6 Hz, 1H), 7.89 – 7.80 (m, 2H), 7.80-7.80 (d, *J* = 7.3 Hz, 1H), 7.23 (d, *J* = 9.1 Hz, 2H), 7.01 (d, *J* = 2.1 Hz, 2H), 6.92 (dd, *J* = 9.2, 2.1 Hz, 2H), 3.95 (t, *J* = 6.2 Hz, 2H), 2.17 (t, *J* = 7.4 Hz, 2H), 1.44 (p, *J* = 7.6 Hz, 2H), 1.36–1.20 (m, 2H), 1.14–0.95 (m, 2H); ^13^C NMR (100 MHz, CD_3_OD) δ 175.8, 173.7, 165.3, 161.0, 158.4, 133.3, 132.7, 131.6, 131.0, 130.4, 130.3, 130.1, 120.8, 115.8, 102.6, 65.3, 33.2, 27.7, 25.1, 24.2; HRMS (ESI^+^) m/z calc’d for C_26_H_23_O_7_ [M + H]^+^: 447.1438, found: 447.1440.



A 25 mL round-bottom flask was charged with **3** (0.23 g, 0.5 mmol, 1.0 equiv), 1-hydroxypyrrolidine-2,5-dione (67.4 mg, 1.2 mmol, 1.2 equiv), DMAP (1.3 mg, 0.05 mmol, 0.1 equiv), DCC (0.21 g, 2.0 mmol, 2.0 equiv) and 10 mL of DCM. The mixture was stirred at 30 °C for 4 h, and then the solvent was removed under reduced pressure. The crude residue was purified by silica gel chromatography (DCM: MeOH = 10: 1) to give **4** (0.24 g, 87%, orange-yellow solid). ^1^H NMR (400 MHz, CDCl_3_) δ 8.26 (dd, *J* = 7.8, 1.6 Hz, 1H), 7.78–7.59 (m, 2H), 7.31 (dd, *J* = 7.3, 1.5 Hz, 1H), 6.95 (d, *J* = 9.2 Hz, 2H), 6.82 (d, *J* = 2.1 Hz, 2H), 6.74 (dd, *J* = 9.3, 2.2 Hz, 2H), 3.96 (t, *J* = 6.1 Hz, 2H), 2.45 (t, *J* = 7.4 Hz, 2H), 1.59–1.47 (m, 2H), 1.36–1.21 (m, 6H), 1.09– 0.96 (m, 2H); ^13^C NMR (100 MHz, CDCl_3_) δ 174.8, 169.7, 168.3, 165.6, 157.3, 153.5, 133.9, 132.7, 131.5, 130.6, 130.42, 130.35, 129.8, 122.1, 115.4, 104.0, 65.3, 30.5, 27.9, 25.6, 25.0, 24.0; HRMS (ESI+) m/z calc’d for C_30_H_26_NO_9_ [M+Na]^+^: 544.1602, found: 544.1602.

The TNMD BRICHOS was dissolved in PBS and incubated with compound 4 overnight at 4 °C in the dark. The next day, an excess of NH₄Cl was added to terminate the reaction. The resulting mixture was concentrated using an ultrafiltration tube (15 kDa, Merck) and repeatedly washed with PBS until the excess compound 4 was removed.

### Analysis of blood-brain barrier (BBB) passage in TNMD BRICHOS

To test the ability of TNMD BRICHOS to pass through the BBB, three-month-old C57BL/6J mice received intravenous injections of 10 mg/kg fluorescein-labeled TNMD BRICHOS or PBS via the tail vein. One hour post-injection, the mice were perfused to wash out residual TNMD BRICHOS from the blood vessels. Images were captured via a small animal imaging system to evaluate whether TNMD could cross the blood-brain barrier.

To further determine the localization of TNMD BRICHOS inside the brain, the frozen mouse brain tissues were cut at a thickness of 20 μm and subjected to immunofluorescence staining. Briefly, the sections were immersed in blocking buffer (1% goat serum, 1% BSA, 0.3% Triton-X100 in 0.01 M PBS) at room temperature for 30 min, and then washed three times with PBST (0.3% Triton X-100 in 0.01 M PBS). Then the sections were incubated with primary antibodies at 4 °C overnight. After the samples were washed with PBST three times, Alexa Fluor 594-conjugated secondary antibodies were applied and incubated at room temperature for 2 h. The sections were then stained with DAPI solution for 5 min. The images were captured using a fluorescence microscope.

### Behavioral test

#### Morris water maze (MWM) test

The MWM procedure was performed as described previously[50]. Visible training was provided on the first day of the experiment. A colored flag was placed on the platform as a conspicuous marker. The mice were individually placed into the water maze, and their movement trajectories were recorded. The primary observed parameters on this day included the swimming speed of the mice and the time the mice were required to reach the platform. From the second to the fifth days of the experiment, the mice underwent platform-finding training. The flag on the platform was removed, and the water level was increased to cover the platform surface. The platform position was also adjusted to ensure that it was different from that on the first day. The mice were placed into the water maze in a random order. If a mouse reached the platform independently, the time taken was recorded. If a mouse failed to reach the platform within 60 s, it was guided to the platform and allowed to stay for 15 s. On the sixth day, the platform was removed for the probe trial, and the mice were again placed into the water maze in a random order. The key parameters recorded during this phase were the time spent and the distance traveled by the mice in the quadrant where the platform had originally been located to assess their spatial memory retention.

#### Y-maze test

The Y-maze consists of three arms of equal length, with each angle between the arms being 120°. Different colored and shaped stickers were placed at the end of each arm to help the mice discern their orientation. A camera was positioned above the Y-maze and connected to ANY-Maze behavioral tracking software to record the movement trajectories of the mice. Each mouse was placed in the center of the Y-maze. Its walking trajectory within the maze was recorded for 10 min. A complete alternation is defined as the mouse sequentially entering each of the three arms of the Y-maze once without repetition. The total number of arm entries made by the mouse was recorded. The number of “major alternations” is calculated as (total arm entries-2). The spontaneous alternation behavior was calculated as (total alternations/major alternations) × 100%. Before the next mouse was tested, the Y-maze was cleaned and sprayed with alcohol to eliminate odors from the previous mouse.

### Statistical analysis

Statistical analysis was performed with GraphPad Prism 10.2. The data are shown as the means ± SD. Statistical comparisons among three or more groups were conducted after normality analysis and homogeneity of variance test. For data conforming to a normal distribution with homogeneous variances, one-way ANOVA followed by Tukey’s multiple comparisons test was performed. For data not conforming to a normal distribution but with homogeneous variances, Brown - Forsythe ANOVA followed by Dunnett’s T3 multiple comparisons test was applied.

Kruskal-Wallis and Dunn’s multiple comparisons test were used to analyze the ratios among groups. Two-way ANOVA with Dunnett’s multiple comparisons test was used for the time-course studies. *P*-values less than 0.05 were considered significant. Sample size was determined based on similar studies in this field, and there were no data exclusions.

## Supplementary information

Supplementary Figure legends

Supplementary Table 1

Supplementary Figure 1

Supplementary Figure 2

Supplementary Figure 3

Supplementary Figure 4

Original Data

## Data Availability

The authors declare that all data supporting the findings of this study are available within the article. The datasets generated during and analyzed during the current study are available from the corresponding author on reasonable request.

## Code availability

The molecular docking for protein-protein interactions was modeled in AlphaFold3 (https://alphafoldserver.com). For each prediction, 5 results were generated and we chose the model with highest iPTM. The structural modeling was generated using Schrodinger Maestro 2025 (Schrödinger Release 2025-4: Maestro, Schrödinger, LLC, New York, NY, 2025) and PyMOL 2.5 (The PyMOL Molecular Graphics System, Version 2.5 Schrödinger, LLC.)[51, 52].
