# Supplementary material for: TNMD BRICHOS domain attenuates tau pathology and memory deficits in a mouse model of tauopathy
Source: Cell Death Dis. 2026 Apr 24;17(1):542. doi: 10.1038/s41419-026-08749-3 (PMC13237185; doi:10.1038/s41419-026-08749-3)
Supplement: Supplementary file 1 — Supplementary Figure legends [file 41419_2026_8749_MOESM1_ESM.docx]

**Supplementary Figure legends**

**Supplementary Figure 1.** **Molecular docking between Tau RD and BRICHOS.**

(**A**-**C**) Predicted interaction interface between ITM2B (A), TNMD (B), OAF BRICHOS domain (C) and tau RD, modelled in AlphaFold3. Blue represents BRICHOS, while pink represents Tau RD. The right panel is a partial enlargement of the left panel, displaying the interaction interface between BRICHOS and Tau RD. The green dotted lines represent pi-pi interactions. The yellow dotted lines denote hydrogen bonds. The purple dotted lines signify ionic bonds. E, Glutamic acid; F, Phenylalanine; G, Glycine; H, Histidine; I, Isoleucine; K, Lysine; L, Leucine; M, Methionine; N, Asparagine; P, Proline; Q, Glutamine; R, Arginine; S, Serine; T, Threonine; V, Valine; W, Tryptophan; Y, Tyrosine.

**Supplementary Figure 2. BRICHOS can enter the cells**

(**A**) Recombinant His-tagged BRICHOS was added to the cell culture medium of HEK293 Tau RD-GFP cells at a final concentration of 0.25 μg/ml and incubated for 48 h. The cells were immunostained with anti-His antibody. Shown are the GFP signals (green) and the His signals (red). Scale bar = 75 μm. (**B**) Primary neurons were incubated with recombinant His-tagged BRICHOS at a final concentration of 0.25 μg/ml and then immunostained with anti-MAP2 (red) and anti-His antibodies (green). Scale bar = 75 μm.

**Supplementary Figure 3. TNMD BRICHOS passes the blood-brain barrier.**

Wild-type mice were administered fluorescently labeled TNMD BRICHOS (10 mg/kg) via the tail vein. One hour later, the mice were perfused with PBS to wash out residual TNMD BRICHOS from the blood vessels. (**A**) Florescence images showing the presence of TNMD BRICHOS (green) in the hippocampus dentate gyrus (DG), CA2, CA3, entorhinal cortex (Ec) and prefrontal cortex (PFC). The nuclei were stained with DAPI (blue). Scale bar = 100 μm. (**B**) Florescence images showing the co - localization of TNMD BRICHOS (green) and neuronal marker MAP2 (red). Scale bar = 25 μm.

**Supplementary Figure 4. Amino acid sequence alignment of the BRICHOS domains of ITM2B, TNMD and OAF.**

The amino acid sequences of the BRICHOS domains of ITM2B, TNMD, and OAF were aligned using DNAMAN software. Dark blue indicates the identical parts among all three sequences, and light blue indicates the parts that are identical between any two of the three sequences.
