# Supplementary material for: TNMD BRICHOS domain attenuates tau pathology and memory deficits in a mouse model of tauopathy
Source: Cell Death Dis. 2026 Apr 24;17(1):542. doi: 10.1038/s41419-026-08749-3 (PMC13237185; doi:10.1038/s41419-026-08749-3)
Supplement: Supplementary file 2 — Supplementary Table 1 [file 41419_2026_8749_MOESM2_ESM.docx]

| **The primers are designed as follows for synthesizing Flag-tagged BRICHOS.** | | |
| --- | --- | --- |
| Name | Forward primer | Reverse primer |
| ITM2A-BRICHOS | ggaattcaagcttggtaccgccaccat | tgcggccgcctaaaagctctttctgttgttacacag |
| ITM2B-BRICHOS | ggaattcaagcttggtaccgccaccat | tgcggccgcctacagcttataggtttccttatcat |
| ITM2C-BRICHOS | ggaattcaagcttggtaccgccaccat | tttgcggccgcctacagccggtaggtgtc |
| GKN1-BRICHOS | ggaattcaagcttggtaccgccaccat | tttgcggccgcctaggccatgtaggtggggat |
| GKN2-BRICHOS | ggaattcaagcttggtaccgccaccat | tgcggccgcctacttgtagagggggatgt |
| TNMD-BRICHOS | ggaattcaagcttggtaccgccaccat | tgcggccgcctagatccagtacattgtcacgt |
| CNMD-BRICHOS | ggaattcaagcttggtaccgccaccat | ttgcggccgcctacagccagaagataggcagat |
| proSP-C-BRICHOS | ggaattcaagcttggtaccgccaccat | tgcggccgcctagatgtagtacagaggcacct |
| BRICD5-BRICHOS | ggaattcaagcttggtaccgccaccat | tttgcggccgcctaggcccagtagataggtgtt |
| OAF-BRICHOS | ggaattcaagcttggtaccgccaccat | tttgcggccgcctatctggtgtagatggcgt |
|  |  |  |
|  |  |  |
| **The primers are designed as follows for expressing the BRICHOS protein in prokaryotes** | | |
|  | Forward primer | Reverse primer |
| ITM2B-BRICHOS | acgcgtcgacacagaccatcgaggaaaacatta | atttgcggccgccagcttataggtttccttatcat |
| TNMD-BRICHOS | acgcgtcgacatccaacggcgagaagaa | atttgcggccgcgatccagtacattgtcacgtt |
| OAF-BRICHOS | acgcgtcgacacctgctgaactgagagt | atttgcggccgctctggtgtagatggcgt |
|  |  |  |
|  |  |  |
|  | **The primers are designed as follows for synthesizing tau fragment** | |
|  | Forward primer | Reverse primer |
|  | aagaaggagatatacatatgggtaccttcatgaaaggactttcaaaggcc | ggcctttgaaagtcctttcatgaaggtacccatatgtatatctccttctt |
