# Supplementary figures and images for: TNMD BRICHOS domain attenuates tau pathology and memory deficits in a mouse model of tauopathy
Source: Cell Death Dis. 2026 Apr 24;17(1):542. doi: 10.1038/s41419-026-08749-3 (PMC13237185; doi:10.1038/s41419-026-08749-3)

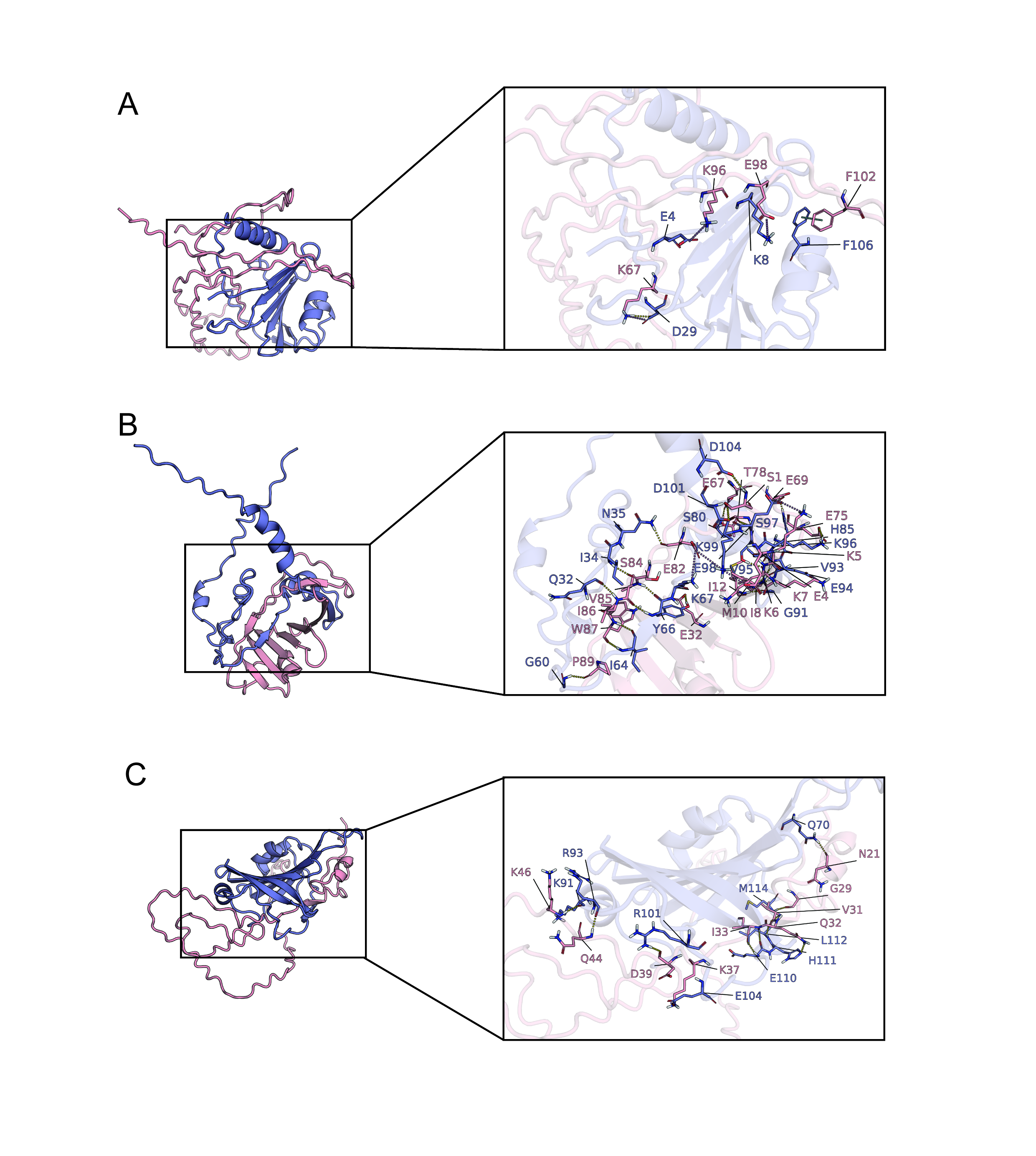

Supplement: Supplementary file 3 — Supplementary Figure 1 [file 41419_2026_8749_MOESM3_ESM.tif]

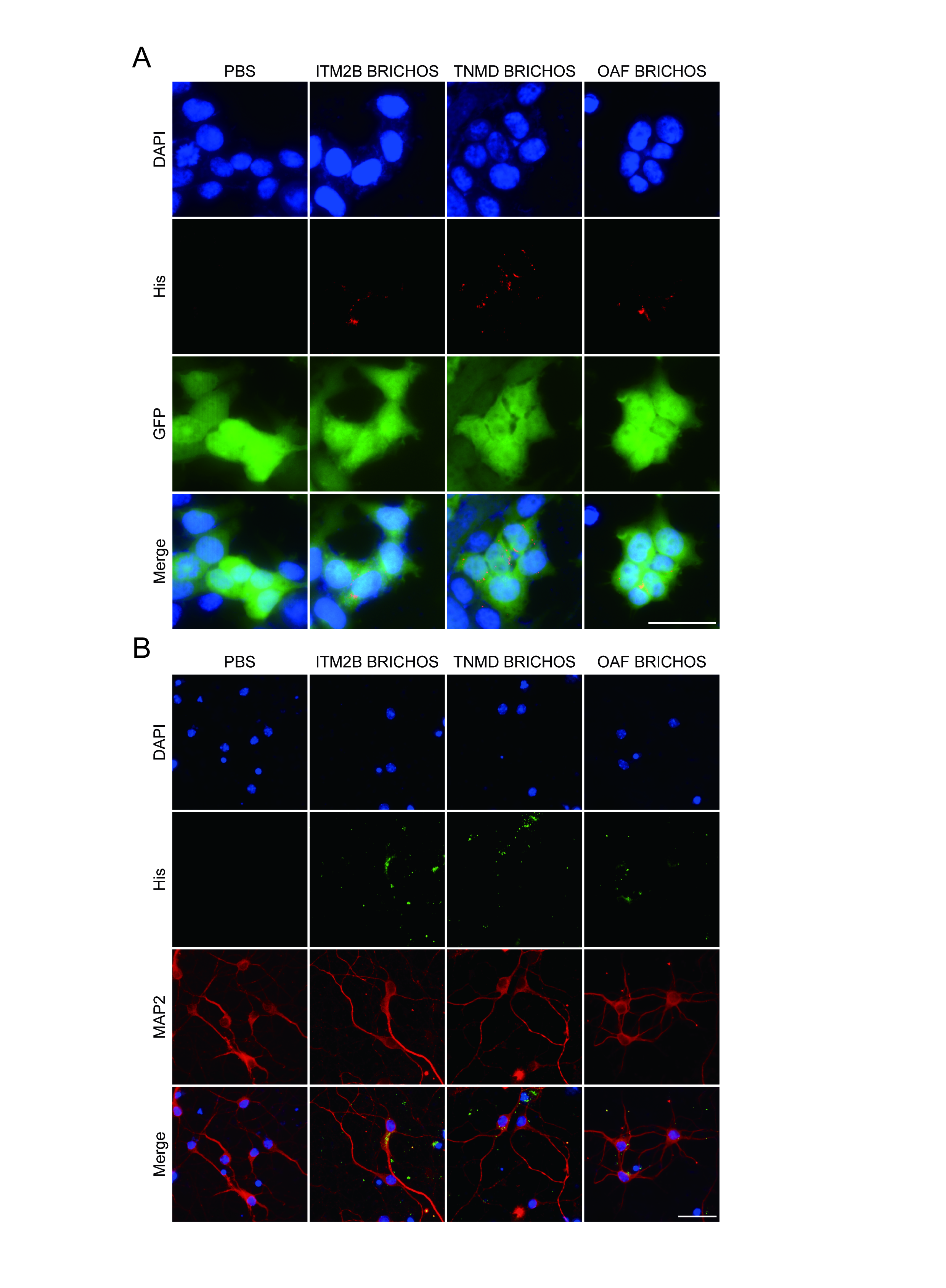

Supplement: Supplementary file 4 — Supplementary Figure 2 [file 41419_2026_8749_MOESM4_ESM.tif]

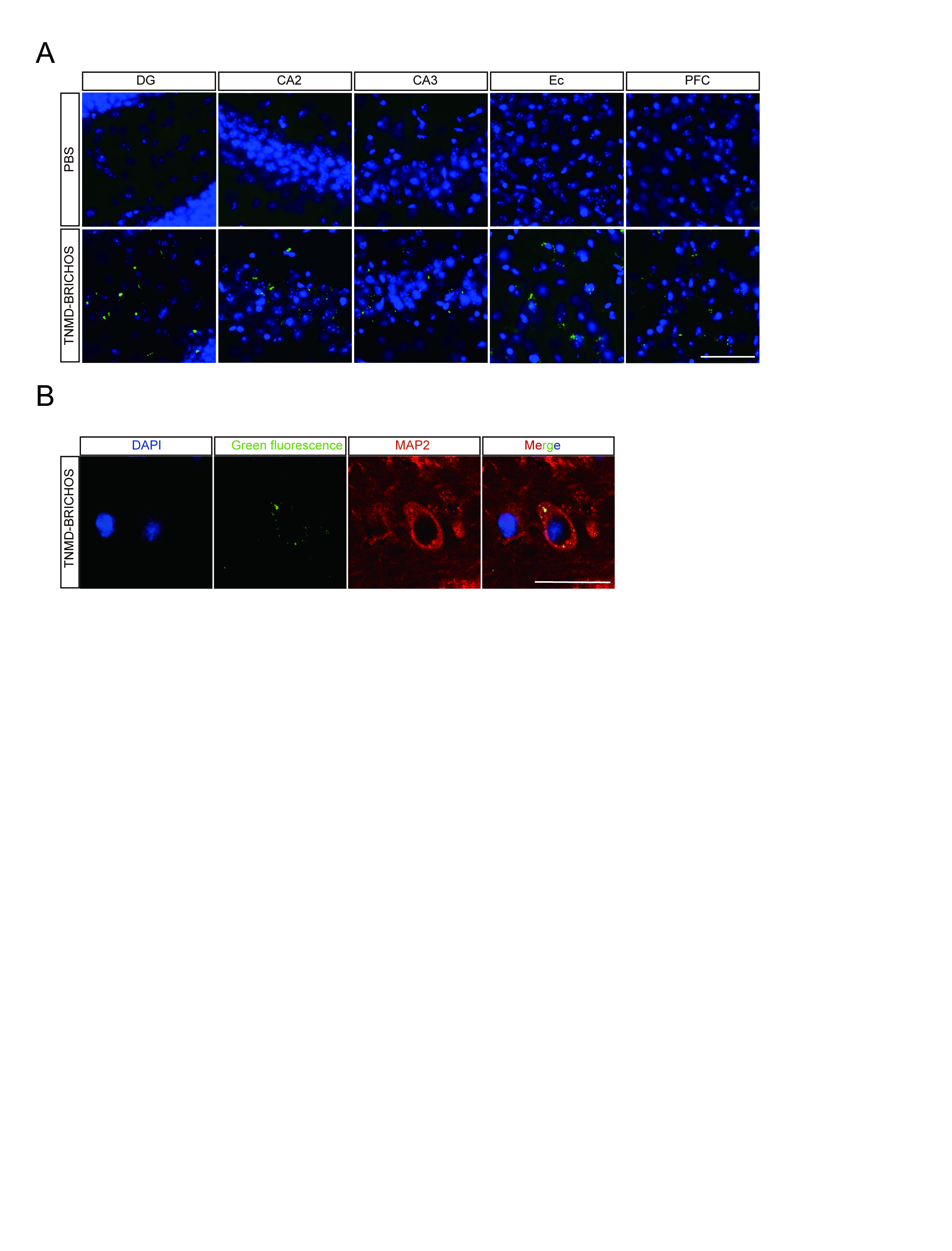

Supplement: Supplementary file 5 — Supplementary Figure 3 [file 41419_2026_8749_MOESM5_ESM.tif]

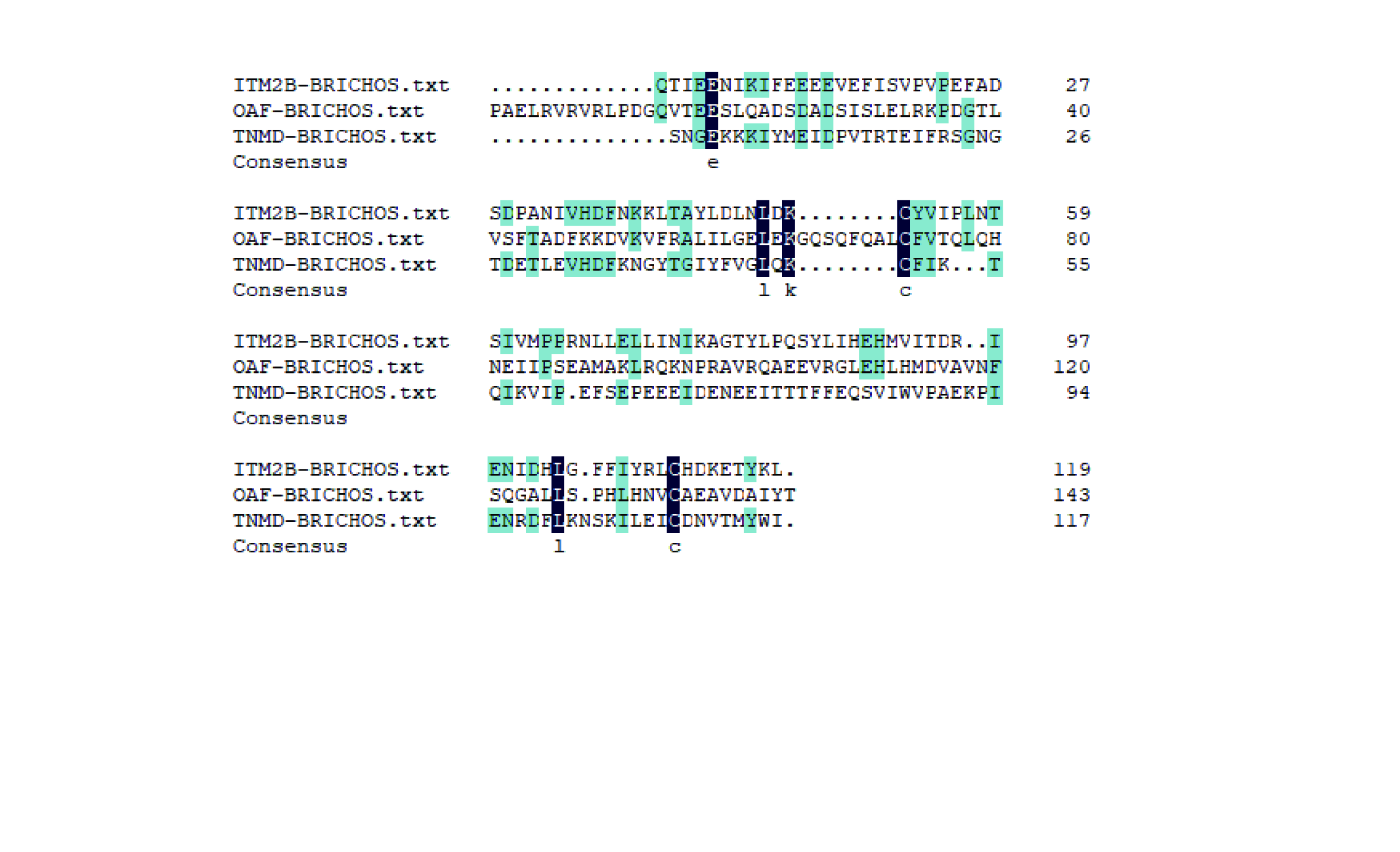

Supplement: Supplementary file 6 — Supplementary Figure 4 [file 41419_2026_8749_MOESM6_ESM.tif]
